# Head Trauma as a Precipitating Factor for Late-onset Leigh Syndrome: a Case Report

**Published:** 2017-01-14

**Authors:** Farzad Ashrafi, Hossein Pakdaman, Mehran Arabahmadi, Behdad Behnam

**Affiliations:** 1 ^Brain Mapping Research Center, Shahid Beheshti University of medical sciences, Tehran, Iran.^

**Keywords:** Leigh disease, craniocerebral trauma, precipitating factors

## Abstract

Leigh syndrome is a severe progressive neurodegenerative disorder with different clinical presentationsthat usually becomes apparent in the first year of life and rarely in late childhood and elderly years. It is causedby failure of mitochondrial respiratory chain and often results in regression of both mental and motor skills and might even lead to death. In some of the inherited neurodegenerative diseases like Alexander disease, head trauma is reported as a trigger for onset of the disease. We present a late onset Leigh syndrome in a 14-year-old girl whose symptoms were initiating following head trauma.

## Introduction

Leigh syndrome (LS) is a rare progressive neurodegenerative disorder, whichusuallymanifests in early childhood. More than 75 gene mutations, related to pyruvate metabolism and mitochondrial respiration, have been found responsible for LS (1). Although LS often occurs in early childhood, there are few reports of late-onset presentation (2). Basal ganglia, cerebellum and brain stem are typically involved by necrotizing changes. The most common manifestations include psychomotor involvement, ataxia, dystonia, seizures, nystagmus, ophthalmoplegia, and respiratory distress (3, 4). This syndrome can present with a variety of clinical presentations, progression and prognosis (5). However, all previous cases have been reported without any trigger before the onset of symptoms. In this report, we present a 14-year-old girl who initially developed seizure and ataxia following head trauma, and finally the diagnosis was proved based on manifestations, characteristic imaging findings and dramatic response to mitochondrial cocktail. 

## Case presentation:

The patient was a 14-year-old girl of consanguineous parents admitted to our emergency department with complaint of respiratory distress. 

**Figure 1:**
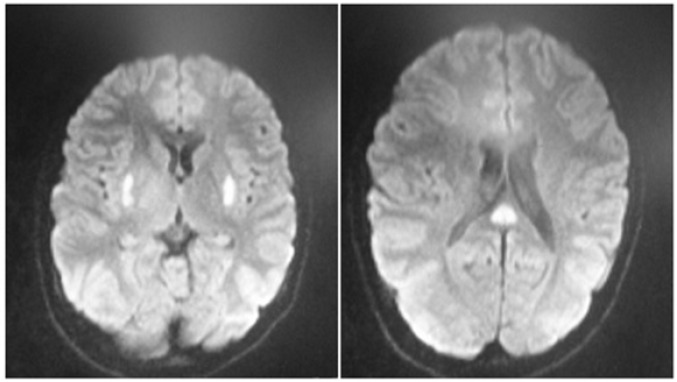
Bilateral basal ganglia hyper-intensities on brain magnetic resonance imaging (MRI

**Figure 2:**
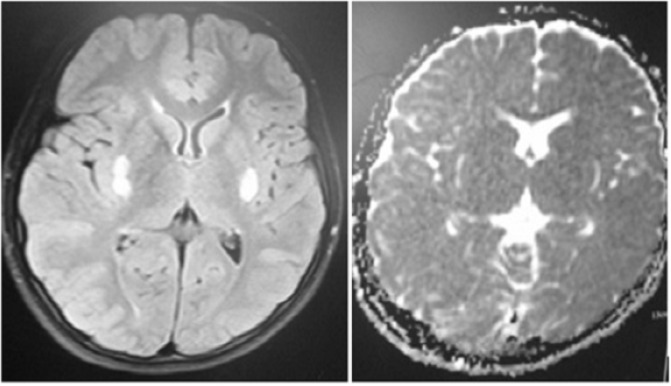
Restriction of the lesions on diffusion weighted (DW) brain magnetic resonance imaging(MRI

**Figure 3:**
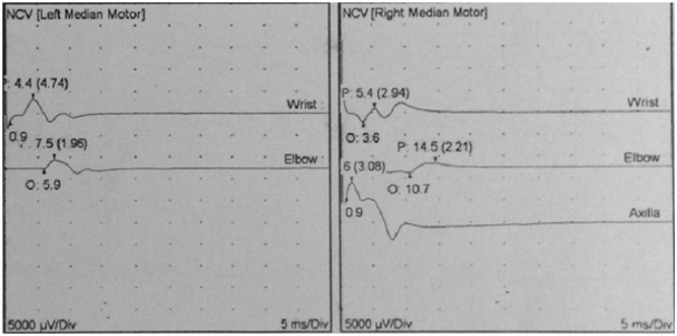
Reduced amplitude of the right and left median motor nerves on nerve conduction velocity (NCV

She had no family history of neurological disorders and had just one 18-year-old sister who was in excellent health. She had no significant past medical history except mild bilateral hearing loss. Her problem first started with a generalized tonic clonic seizure following head trauma during sportsclassin school 9 months ago. According to her parents’ report, she had about one minute postictal phase after her first episode of seizure. She was admitted to hospital and tooklamotrigin and phenytoin that were discontinued gradually by improvements of her symptoms.

Two month later, she developed ataxia, difficulties with walking and generalized weakness. At that time, her neurological examinations revealed no abnormalities except spastic tone of muscles and upward plantar reflex. 

Her blood biochemistry, urine analysis, and cerebro-spinal fluid (CSF) study were all within normal limits except for increased serum pyruvate level (2 mg/dl, normal: 0.3 - 0.7) and elevated CSF lactate (3.2 mmol/L; normal: 1.1-2.3). In addition, urine copper, serum copper and ceruloplasminlevels were in normal range. 

Brain magnetic resonance imaging (MRI) showed symmetric bilateral hypersignal lesions in basal ganglia accompanied by isosignal lesions in splenium and corpus callossum (Figure1). Basal ganglia lesions were negative on diffusion weighted (DW) while splenium and corpus callossum were positive, restricted with low apparent diffusion coefficient (Figure 2).

Magnetic resonance spectroscopy also reported an abnormal hyper intensity with near symmetrical configuration in periventricular region, brain stem and medulla with lactate rise and diffusion restriction compatible with mitochondrial disorders or glutaricaciduria.Electromyography/nerve conduction velocity (EMG/NCV) revealed axonal polyneuropathy with neurogenic changes without active denervation (Figure 3). Ophthalmology consultation was negative for retinitis pigmentosaand Kayser–Fleischer ring. With the impression of metabolic disease, she was treated with Co Q10, Vitamin B1, L-Carnitine and B-complex (mitochondrial cocktail) and dramatically responded to treatment and her neurological conditions improved.  

She was discharged without any complaint and suggested to come back to clinic with genetic study report, complete treatment and follow up.

Two weeks later she presented to emergency room with respiratory distress. On admission, she was ill and not oriented. Her blood pressure was 140/70 mmHg, pulse rate of 141/minute, respiratory rate of 45/minute, temperature 37.3°Cand O_2_ saturation 89%. Her arterial blood gas analysis revealed pH of 7.40, PaO_2_ of 60.2, PaCO_2_ of 27 and HCO_3_ of 16. General examinations were normal except tachypneic breathing with subcostal retraction.  Her neurological examination was remarkable for left eye deviation (abducent nerve paresia) and generalized weakness. Her lower limb muscular force was 3/5. She was intubated and supportive therapy was begun for her.She had cardiac arrest and died despite cardiopulmonary resuscitation.

Three days later, her genetic study revealed homozygote mutation (35 del G) in evaluation of entire coding regions of GJB2 and confirmed the diagnosis of LS. A diagnosis of late-onset LS was made based on radiological and response to mitochondrial cocktail. 

## Discussion

LS is categorized as a mitochondrial encephalomyelopathies. Defect in enzyme pathway for respiratory metabolism has been known as an underlying factor (6). In this case, the most interesting aspect was the head trauma as a possible precipitating factor in developing seizure and other symptoms in a previously normal child that finally led to diagnosis of LS. Up to now, few cases have been reported who developed neurological disorders with underlying gene mutation following head trauma. In 2010, Namekawa et al, reported an adult onset of Alexander disease in a 60 year old man whose symptoms initiated after head trauma (7). Furthermore, Hayashi et al.presented a 34-year-old man who developed a gradual visual problem about 6 months after an accident and was finally diagnosed with Leber's hereditary optic neuropathy (8). 

Moreover, association between traumatic brain injury and mitochondrial pathway defect has been shown previously (9). Our case, together with previous case reports raised the possibility of trauma being a precipitating factor in developing or progression of mitochondrial disorders.

## Conclusion:

This case showed the possible role of triggers such as head trauma in developing LS in patients with underlying genetic defect.
